# *Ex vivo *susceptibility of *Plasmodium falciparum *isolates from Dakar, Senegal, to seven standard anti-malarial drugs

**DOI:** 10.1186/1475-2875-10-310

**Published:** 2011-10-20

**Authors:** Bécaye Fall, Silmane Diawara, Kowry Sow, Eric Baret, Bakary Diatta, Khadidiatou B Fall, Pape S Mbaye, Fatou Fall, Yaya Diémé, Christophe Rogier, Boubacar Wade, Raymond Bercion, Bruno Pradines

**Affiliations:** 1 Laboratoire d'étude de la chimiosensibilité du paludisme, Fédération des laboratoires, Hôpital Principal de Dakar, Dakar, Sénégal; 2 Unité de parasitologie - Unité de recherche sur les maladies infectieuses et transmissibles émergentes - UMR 6236, Institut de recherche biomédicale des armées, Allée du Médecin-colonel Jamot, Parc le Pharo, BP 60109, 13262 Marseille Cedex 7, France; 3 Service de réanimation médicale, Hôpital Principal de Dakar, Dakar, Sénégal; 4 Service de pathologie infectieuse, Hôpital Principal de Dakar, Dakar, Sénégal; 5 Département de médecine interne et spécialités médicales de pathologie tropicale, Hôpital Principal de Dakar, Dakar, Sénégal; 6 Service interne d'hépato-gastroentérologie, Hôpital Principal de Dakar, Dakar, Sénégal; 7 Chefferie, Hôpital Principal de Dakar, Dakar, Sénégal; 8 Centre National de référence du Paludisme, Marseille, France

## Abstract

**Background:**

As a result of widespread chloroquine and sulphadoxine-pyrimethamine resistance, artemisinin-based combination therapy (ACT) (which includes artemether-lumefantrine and artesunate-amodiaquine) has been recommended as a first-line anti-malarial regimen in Senegal since 2006. Since then, there have been very few reports on the *ex vivo *susceptibility of *Plasmodium falciparum *to anti-malarial drugs. To examine whether parasite susceptibility has been affected by the widespread use of ACT, the *ex vivo *susceptibility of local isolates was assessed at the military hospital of Dakar.

**Methods:**

The *ex vivo *susceptibility of 93 *P. falciparum *isolates from Dakar was successfully determined using the *Plasmodium *lactate dehydrogenase (pLDH) ELISA for the following drugs: chloroquine (CQ), quinine (QN), mefloquine (MQ), monodesethylamodiaquine (MDAQ), lumefantrine (LMF), dihydroartemisinin (DHA) and doxycycline (DOX).

**Results:**

After transformation of the isolate IC_50 _in ratio of IC_50 _according to the susceptibility of the 3D7 reference strain (isolate IC_50_/3D7 IC_50_), the prevalence of the *in vitro *resistant isolates with reduced susceptibility was 50% for MQ, 22% for CQ, 12% for DOX, 6% for both QN and MDAQ and 1% for the drugs LMF and DHA. The highest significant positive correlations were shown between responses to CQ and MDAQ (*r *= 0.569; *P *< 0.0001), LMF and QN (*r *= 0.511; *P *< 0.0001), LMF and DHA (*r *= 0.428; *P *= 0.0001), LMF and MQ (*r *= 0.413; *P *= 0.0002), QN and DHA (*r *= 0.402; *P *= 0.0003) and QN and MQ (*r *= 0.421; *P *= 0.0001).

**Conclusions:**

The introduction of ACT in 2002 has not induced a decrease in *P. falciparum *susceptibility to the drugs DHA, MDAQ and LMF, which are common ACT components. However, the prevalence of *P. falciparum *isolates with reduced susceptibility has increased for both MQ and DOX. Taken together, these data suggest that intensive surveillance of the *P. falciparum in vitro *susceptibility to anti-malarial drugs in Senegal is required.

## Background

During the past 20 years, many strains of *Plasmodium falciparum *have become resistant to chloroquine and other anti-malarial drugs [1]. The emergence and spread of drug-resistant parasites has generated an urgent need for the development of novel anti-malarial drugs. One strategy for reducing malaria prevalence is the use of drugs in combination. Drug combinations protect each component drug from the development of resistance and reduce the overall transmission of malaria [2]. Since 2001, more than 60 countries have officially adopted artemisinin-based combination therapy (ACT) for the treatment of falciparum malaria; moreover, ACT is now the official first-line treatment against malaria in Africa [3]. In ACT, the artemisinin derivative causes rapid and effective reduction of parasite biomass and gametocyte carriage, while the partner drug, which has an extended duration of activity, achieves effective clinical and parasitological cure. However, the clinical failures, or at least the extended parasite clearance times associated with ACT have been described in Cambodia [4-7]. In addition, prior therapy with ACT containing amodiaquine has selected for *P. falciparum *isolates with reduced response to monodesethylamodiaquine, thereby suggesting that amodiaquine-containing regimens may have rapidly reduced efficacy in Africa [8].

Dakar, the capital city of Senegal, has an urban population of approximately 1.1 million and a suburban population of 2.3 million; the city covers the majority of the Cap-Vert Peninsular. Malaria is transmitted in Dakar and its surrounding suburbs, with a human biting rate ranging from 0.1 to 43.7 bites per person per night, according to different districts and entomological inoculation rates (EIR) ranging from 0 to 16.8 infected bites per person per year [9,10]. Malaria transmission rates are associated with environmental factors, such as vegetation and built-up area [11]. Malaria accounted for 25.9% of the total cases observed in the Infectious Disease Clinic in Dakar between 2001 and 2003. Of those cases, 65.6% presented as severe forms [12]. In 2004, malaria attacks accounted for 13.7% (2977 cases) of all consultations at the Dispensaire St Martin in Dakar, which increased from October to December [13].

Since 2002, ACT has been routinely and widely used in Senegal for managing uncomplicated malaria. ACT is delivered free-of-charge in health centres. Together, artemether-lumefantrine and artesunate-amodiaquine were the ACT recommended as the first-line anti-malarial regimen since it became available in the public system in 2006. Since then, more than 1.5 million treatments have been administered [14]. In 2002-2003, an open randomized study with the drug Artequin^® ^(artesunate-mefloquine) showed an excellent clinical and parasitological response rate of 100% on day 28 [15]. In 2008, in Kaolack (200 km south-east of Dakar), a study showed that Artequin was efficacious in paediatric patients with a cure rate of 96.2% in intention-to-treat analysis and was as well tolerated as artemether-lumefantrine [16]. Artesunate-amodiaquine associated cure rates were > 99.3% in Mlomp and Keur-Socé when administered either as a single daily intake or two daily intakes [17]. The fixed-dose combination of artesunate-amodiaquine (ASAQ) shows a cure rate > 98.5% [18]. The cure rates were 100% in both populations experiencing a second and third episode of uncomplicated malaria following treatment with ASAQ [14].

Since the introduction of ACT in Senegal, there have been very few reports on the *ex vivo *susceptibility of *P. falciparum *to anti-malarial drugs. To examine whether parasite susceptibility has been affected by the widespread use of ACT, an *ex vivo *susceptibility study was conducted with local isolates obtained at the military hospital of Dakar. The new term '*ex vivo *susceptibility' is used to describe studies on fresh isolates, while the term '*in vitro *susceptibility' should now refer to studies on strains of the parasite, which have been either kept in culture for at least two to three generations or which have been cryo-preserved. The malaria isolates obtained from patients at the Hôpital Principal de Dakar were assessed for susceptibility to chloroquine (CQ), quinine (QN), monodesethylamodiaquine (MDAQ), the active metabolite of amodiaquine, mefloquine (MQ), lumefantrine (LMF), dihydroartemisinin (DHA), the active metabolite of artemisinin derivatives and doxycycline (DOX).

## Methods

### *Plasmodium falciparum *isolates

In total, 188 patients with malaria were recruited from 14 October 2009 to 19 January 2010 at the Hôpital Principal de Dakar. Venous blood samples were collected in Vacutainer^® ^ACD tubes (Becton Dickinson, Rutherford, NJ, USA) prior to patient treatment; blood samples were used to test drug susceptibility within less than 12 hours (h) of collection. Informed verbal consent was obtained from patients and/or their parents before blood collection. Susceptibility tests were performed on the same blood samples used for malaria diagnosis. The study was reviewed and approved by the ethical committee of Hôpital Principal de Dakar.

Thin blood smears were stained using a RAL^® ^kit (Réactifs RAL, Paris, France) and were examined to determine *P. falciparum *density and to confirm monoinfection. Parasitized erythrocytes were washed three times in RPMI 1640 medium (Invitrogen, Paisley, UK) buffered with 25 mM HEPES and 25 mM NaHCO_3_. If parasitaemia exceeded 0.8%, infected erythrocytes were diluted to 0.4% with uninfected erythrocytes (human blood type A+) and re-suspended in RPMI 1640 medium supplemented with 10% human serum (Abcys S.A. Paris, France), for a final haematocrit of 1.5%.

### Drugs

CQ, QN, DHA and DOX were purchased from Sigma (Saint Louis, MO, USA). MDAQ was obtained from the World Health Organization (Geneva, Switzerland), MQ was purchased from Roche (Paris, France) and LMF was purchased from Novartis Pharma (Basel, Switzerland). QN, MDAQ, MQ, DHA and DOX were dissolved first in methanol and then diluted in water to final concentrations ranging from 5 nM to 3200 nM for QN, 1.56 nM to 1000 nM for MDAQ, 3.2 nM to 400 nM for MQ, 0.1 nM to 100 nM for DHA and 0.1 μM to 502 μM for DOX. CQ was re-suspended and diluted in water to final concentrations ranging from 5 nM to 3200 nM. LMF was re-suspended and diluted in ethanol to obtain final concentrations ranging from 0.5 nM to 310 nM.

The batches of plates were tested and validated on the CQ-susceptible 3D7 strain (West-Africa) and the CQ-resistant W2 strain (Indochina) (MR4, Virginia, USA) in 3 to 6 independent experiments using both the standard 42-h ^3^H-hypoxanthine uptake inhibition method [19] in controlled atmospheric conditions in an incubator (5% CO_2_, 10% O_2 _and 85% N_2_) and the *Plasmodium *lactate dehydrogenase (pLDH) ELISA [20,21] with the same conditions described in the paragraph below. The two strains were synchronized twice with sorbitol before use [22], and clonality was verified every 15 days using PCR genotyping of the polymorphic genetic markers *msp1 *and *msp2 *and using microsatellite loci [23,24] and each year by an independent laboratory from the Worldwide Anti-malarial Resistance Network (WWARN).

### *Ex vivo *assay

For the *in vitro *isotopic microtests, 200 μl of synchronous parasitized red blood cells (final parasitaemia, 0.5%; final haematocrit, 1.5%) was aliquoted into 96-well plates pre-dosed with anti-malarial drugs. The plates were incubated in a sealed bag for 42 h at 37°C with the atmospheric generators for capnophilic bacteria Genbag CO2^® ^at 5% CO_2 _and 15% O_2 _(BioMérieux; Marcy l'Etoile, France) [25]. After thawing the plates, haemolysed cultures were homogenized by vortexing the plates. Both the success of the drug susceptibility assay and the appropriate volume of haemolysed culture to use for each assay were determined for each clinical isolate during a preliminary pLDH ELISA. This pre-test and the subsequent ELISAs were performed using the commercial kit (ELISA-Malaria antigen test, ref 750101, DiaMed AG, Cressier s/Morat, Switzerland) as previously described [20]. The optical density (OD) of each sample was measured with a spectrophotometer (Multiskan EX, Thermo Scientific, Vantaa, Finland).

The concentration at which the drugs were able to inhibit 50% of parasite growth (IC_50_) was calculated with the inhibitory sigmoid Emax model with estimation of the IC_50 _through non-linear regression using a standard function of the R software (ICEstimator version 1.2) [26]. IC_50 _values were validated only if the OD ratio (OD at concentration 0/OD at concentration max) was superior to 1.8 and the confidence interval ratio (upper 95% confidence interval of the IC_50 _estimation/lower 95% confidence interval of the IC_50 _estimation) was inferior to 2.0 [26].

### Statistical analysis

IC_50 _values were analysed after logarithmic transformation and expressed as the geometric mean of the IC_50 _and the confidence interval 95% (CI95%). Assessment of cross-resistance between the seven anti-malarial drugs was measured by pair-wise correlation of IC_50 _values of all isolates and estimated by the Pearson coefficient of correlation (*r*) and the coefficient of determination (*r^2^*). The cut-off values for *in vitro *resistance, or reduced susceptibility, were re-evaluated under experimental conditions induced by the use of the atmospheric generators for capnophilic bacteria Genbag CO2 by regression. This experiment was performed to compare the prevalence of isolates with *in vitro *resistance, or reduced susceptibility, tested in Genbag conditions [25] with the prevalence of isolates using the standard 42-h ^3^H-hypoxanthine uptake inhibition method in controlled atmospheric conditions in the incubator (5% CO_2_, 10% O_2 _and 85% N_2_) [19] with a cut-off concentration of 100 nM, 80 nM, 150 nM, 10.5 nM, 800 nM, 30 nM and 35 μM for CQ, MDAQ, LMF, DHA, QN, MQ and DOX, respectively. Using the *Plasmodium *lactate dehydrogenase (pLDH) ELISA in Genbag conditions, the values were 77 nM, 61 nM, 115 nM, 12 nM, 611 nM, 30 nM and 37 μM for CQ, MDAQ, LMF, DHA, QN, MQ and DOX, respectively. An IC_50 _ratio (IC_50 _of clinical isolate/mean IC_50 _of 3D7 on the same batch of plates tested in 3 to 6 independent experiments) was calculated for each anti-malarial drug and each isolate in accordance with the guidelines of the WWARN. These ratios were also analysed after logarithmic transformation and expressed as the geometric mean of the ratios and the confidence interval 95% (CI95%). 3D7 ratio cut-offs were estimated for *in vitro *resistance or reduced susceptibility by calculating the ratio of standard threshold at Genbag CO2^® ^conditions/3D7 IC_50 _mean. These values were 5 (CQ), 3 (MDAQ), 5 (LMF), 7 (DHA), 5 (QN), 0.6 (MQ) and 3 (DOX).

## Consent

The study was reviewed and approved by the ethics commission of Hôpital Principal de Dakar.

## Results

One hundred and eighty eight patients were recruited for the study at the Hôpital Principal de Dakar; 147 isolates from these patients were tested *ex vivo*, and 93 isolates were successfully cultured. After validating the IC_50 _[(OD at concentration = 0/OD at concentration max) > 1.8 and (upper 95% confidence interval/lower 95% confidence interval) < 2.0], the following proportion of validated IC_50 _values were 83 for CQ, 83 for MDAQ, 70 for LMF, 88 for DHA, 82 for QN, 88 for MQ and 76 for DOX.

The average parameter estimates for the seven anti-malarial drugs utilized against the *P. falciparum *isolates are given in Table 1. The prevalence of *P. falciparum *isolates with *in vitro *decreased susceptibility to MQ reached 55% (Table 2). Only 22% of the isolates were resistant to CQ. The percent of resistant isolates, or those with decreased susceptibility, were similar using the 3D7 ratio cut-offs.

**Table 1:** *Ex vivo *susceptibility of 93 *Plasmodium falciparum *isolates from Dakar to chloroquine (CQ), monodesethylamodiaquine (MDAQ), lumefantrine (LMF), dihydroartemisinin (DHA), quinine (QN), mefloquine (MQ) and doxycycline (DOX)

		**Isolate IC^50^**				Ratio (Isolate IC^50^ /Mean 3D7 IC^50^)			
		
Drug	No	Mean	CI95%	Min	Max	Mean	CI95%	Min	Max
CQ	83	34.8 nM	26.9-45.0	4.6	1324	2.1	1.6-2.7	0.26	81.7
MDAQ	83	15.7 nM	12.8-19.4	1.5	82.8	0.8	1.6-2.7	0.07	3.9
LMF	79	21.3 nM	17.1-26.6	1.5	150	0.8	0.6-1.0	0.05	5.8
DHA	88	1.6 nM	1.3-1.8	0.1	9.4	0.9	0.7-1.0	0.05	7.3
QN	82	159 nM	128-197	6.4	1291	1.2	1.0-1.5	0.06	8.6
MQ	88	29.8 nM	24.3-36.5	3.0	170	0.5	0.4-0.7	0.05	3.0
DOX	76	11.6 μM	9.5-14.2	0.5	48.1	1.1	0.9-1.4	0.04	4.7

Mean: geometric mean

CI95%: 95% confidence interval

**Table 2:** Prevalence of *in vitro *resistant *Plasmodium falciparum *isolates from Dakar or with reduced susceptibility to chloroquine (CQ), monodesethylamodiaquine (MDAQ), lumefantrine (LMF), dihydroartemisinin (DHA), quinine (QN), mefloquine (MQ) and doxycycline (DOX)

Drug	**Resistant or reduced susceptible isolate IC^50^**			Resistant or reduced susceptible Ratio (isolate IC^50^ /3D7 IC^50^)		
	Cut-off	No	%	Cut-off	No	%
CQ	77 nM	18/83	22	5	18/83	22
MDAQ	61 nM	5/83	6	3	5/83	6
LMF	115 nM	3/79	4	5	1/79	1
DHA	12 nM	0/88	0	7	1/88	1
QN	611 nM	9/82	11	5	5/82	6
MQ	30 nM	48/88	55	0.6	44/88	50
DOX	37 μM	6/76	8	3	9/76	12

The highest significant positive correlations were shown between responses to CQ and MDAQ (*r *= 0.569; *P *< 0.0001), LMF and QN (*r *= 0.511; *P *< 0.0001), LMF and DHA (*r *= 0.428; *P *= 0.0001), LMF and MQ (*r *= 0.413; *P *= 0.0002), QN and DHA (*r *= 0.402; *P *= 0.0003) and QN and MQ (*r *= 0.421; *P *= 0.0001) (Table 3).

**Table 3:** Correlation of *in vitro *responses (Log IC_50_) of 93 isolates of *Plasmodium falciparum *from Dakar to chloroquine (CQ), monodesethylamodiaquine (MDAQ), lumefantrine (LMF), dihydroartemisinin (DHA), quinine (QN), mefloquine (MQ) and doxycycline (DOX)

		CQ	MDAQ	LMF	DHA	QN	MQ	DOX
MDAQ	r	0.569	1					
	p-value	< 0.0001						
LMF	r	0.071	0.004	1				
	p-value	0.5371	0.9732					
DHA	r	0.044	0.307	0.428	1			
	p-value	0.7038	0.0065	< 0.0001				
QN	r	0.205	0.439	0.511	0.402	1		
	p-value	0.0670	< 0.0001	< 0.0001	0.0003			
MQ	r	0.267	0.412	0.413	0.357	0.421	1	
	p-value	0.0180	0.0002	0.0002	0.0008	0.0001		
DOX	r	0.313	0.025	0.306	0.249	0.318	0.390	1
	p-value	0.0105	0.8438	0.0157	0.0347	0.0104	0.0007	

## Conclusions

This report describes the evaluation of the *ex vivo *susceptibility of *P. falciparum *isolates taken from patients in Dakar to seven standard anti-malarial drugs. Analysis of the *in vitro *susceptibility of *P. falciparum *isolates has three important attributes [27]. First, this approach allows one to assay the response of clinical isolates to individual drugs that remain intact and unmodified by important host factors that influence drug efficacy *in vivo*. Second, the progressive decline in drug susceptibility of isolates from the same site may identify incipient resistance in the parasite population. Finally, strains with reduced susceptibilities to anti-malarial drugs can be established in continuous culture, thus providing the material needed to investigate novel molecular mechanisms of resistance. These cultures can also provide material for tests of susceptibility to other anti-malarial agents.

This study represents the first use of the 3D7 ratio to address the requirement of the *in vitro *module of WWARN. The use of this 3D7 ratio could allow for the minimization of bias induced by IC_50 _variations, which can be caused by plate batches, atmospheric conditions and varying methodologies in general. The 3D7 ratio could allow researchers to compare data and monitor the spatial and temporal evolution of resistance to anti-malarial drugs. However, the estimation of the 3D7 ratio cut-off for *in vitro *resistance or reduced susceptibility must be refined by using a large series of 3D7 IC_50_. Nevertheless, the results in terms of prevalence of resistance are relatively similar between the two estimations.

Previous *ex vivo *studies in Dakar and in the Fatick region (Dielmo and Ndiop, 280 km south-east of Dakar) were conducted using the same methodology (42-h ^3^H-hypoxanthine uptake inhibition method in controlled atmospheric conditions 5% CO_2 _and 10% O_2_) [24,28-35], while certain conditions varied from that in the present study (atmospheric generators for capnophilic bacteria Genbag CO2^® ^at 5% CO_2 _and 15% O_2 _and pLDH ELISA). However, the re-evaluation of the cut-off values for *in vitro *resistance or reduced susceptibility in these new experimental conditions induced by the use of the atmospheric generators for capnophilic bacteria Genbag CO2^® ^allows for the comparison of these data with previous data assessed by the standard 42-h ^3^H-hypoxanthine uptake inhibition method in controlled incubator conditions (5% CO_2 _and 10% O_2_) [25].

Surprisingly, the prevalence of isolates with reduced susceptibility to MQ reached 50%. This level has increased since previous studies conducted in Senegal. In Dielmo and Ndiop, the percent of isolates with decreased susceptibility was 22% in 1995 [28] and 15% in 1999 [33,35]. In Dakar, it was 17% in 2001 [36] and 13% in 2002 [24]. Prophylaxis failure with MQ has been previously described in Senegal [37]. MQ is one of the three anti-malarial drugs recommended for travellers as an anti-malarial prophylaxis in Senegal. Clinical trials are in progress to evaluate the efficacy of MQ for intermittent preventive treatment for infants and during pregnancy, while MQ is still used for the treatment of uncomplicated malaria in infants in Dakar. Nevertheless, MQ has been employed relatively infrequently in Africa as compared to Asia. The combination artesunate-mefloquine, which is administered to patients in Asia, is not yet used in Senegal. However, scientific data are not available for MQ monotherapy. A positive correlation was shown between MQ and QN (*r *= 0.421; *r^2 ^*= 0.178) and between the responses to the components of the ACT used in Dakar, LMF (*r *= 0.413; *r^2 ^*= 0.171), MDAQ (*r *= 0.412; *r^2 ^*= 0.170) and DHA (*r *= 0.402; *r^2 ^*= 0.161). However, to suggest common mechanisms of action or resistance for two compounds that could induce cross-resistance, the coefficient of determination must be high. A coefficient of determination of 0.161, 0.170, 0.171 and 0.178 means that only 16.1%, 17.0%, 17.1% and 17.8% of the variation in the response to MQ is explained by the variation in the response to DHA, MDAQ, LMF and QN, respectively. The IC_50 _value of the isolate with the highest DHA IC_50 _(9.37 nM) was 3.22 nM for MQ. Meanwhile, the IC_50 _value of the isolate with the highest MQ IC_50 _(170 nM) was 1.63 nM for DHA. Very few data are available on the *in vitro *decreased susceptibility to MQ and its clinical implications in Africa. It is important to monitor the evolution of *P. falciparum *susceptibility to MQ, to archive suspicious isolates and to correlate clinical outcomes with pharmacokinetic and phenotypic responses and with molecular markers.

As far back as 1988, *in vitro P. falciparum *resistance to CQ was reported in Dakar, and reports of resistance in other regions of the country followed shortly [38]. From 1991 to 1995, parasitological failures were observed in 21% of patients in Pikine and in 23% of patients in another region of Senegal [39]. The *in vitro *resistance to CQ increased from 1995 to 1999 in Dielmo with 32% resistance in 1995 [29] as compared to 49% resistance in 1996 [30], 44% resistance in 1997 [34] and 55% resistance in 1999 [33]. Certain single nucleotide polymorphisms (SNPs), such as the *P. falciparum *chloroquine resistance transporter gene (*pfcrt*) polymorphism K76T, are associated with decreased susceptibility to CQ. Furthermore, this decreased susceptibility to CQ in Dielmo was confirmed by evaluation of the K76T mutation [40]. In Dielmo, the incidence of clinical malaria for patients within seven days of CQ treatment increased from 2.6% in 1995 to 13% in 1999, despite strictly controlled anti-malarial use [40]. A similar increase in *in vitro *resistance was seen in Dakar and the suburb of Pikine with a prevalence of 30% to 31% of strains being resistant to CQ in 2000 [41,42] increasing to 52% after 2002 [24,43]. The prevalence of the K76T mutation, which is associated with CQ resistance, was greater than 50% in both 2001 [41,43] and 2002 [24]. In 2009, in Dakar, only 22% of isolates were found to be resistant *ex vivo *to CQ. These data are consistent with the work of Ndiaye *et al*., who showed that 23% of parasites were resistant to CQ in 2007 in Thies (75 km south-east of Dakar) [44]. This decrease in CQ resistance parallels the withdrawal of CQ drug treatment and the introduction of ACT in 2002 in Senegal. However, in 2003, CQ was still being administered to patients. CQ was found in the urine samples of two nine-year-old children, with a prevalence ranging from 14.5% to 47.5% in children from north Senegal and from 9.0% to 21.4% in children from south Senegal [45]. In 2006, Senegal reported 10.6% CQ use and 9.7% ACT use [46]. Since 2006, more than 1.5 million ACT treatments have been administered in Senegal [14]. A similar reduction in CQ resistance was reported in Malawi after withdrawal of CQ treatment [47]. This observation prompted a CQ *in vivo *study in Malawi five years later, in which CQ was found to be 99% effective [48]. The rapid dissemination of CQ resistance in Dielmo, despite strictly controlled anti-malarial drug use, argues against the re-introduction of CQ in places where the resistant allele has dropped to very low levels following discontinuation of CQ treatment [40].

Encouragingly, only 6% of isolates show *in vitro *reduced susceptibility to MDAQ. This rate is stable with 0% resistance in 1996 and 1999 in Dielmo [29,35] and 5% in Mlomp (Casamance, south-western Senegal) in 2004 [49]. The rates remained low even after the introduction of artesunate-amodiaquine in 2002 in Senegal. A positive correlation was shown between MDAQ and CQ responses (*r *= 0.569; *r^2 ^*= 0.324), which means that 32.4% of the variation in the response to MDAQ is explained by variation in the response to CQ. A study in Dakar and Mlomp from 1996 to 1998 showed that monotherapy with amodiaquine remained effective for treating uncomplicated malaria in areas where CQ resistance was prevalent [50].

Only one isolate exhibited reduced susceptibility to DHA. This result is consistent with previous studies that did not show any parasites resistant to artesunate [24,33,35]. However, Agnamey *et al*. reported that 3% - 23% of isolates had IC_50 _values greater than 15 nM in Mlomp between 2000 and 2004 [50]. High IC_50 _values can also be found for artemisinin with IC_50 _> 30 nM in Dakar [44] and artesunate with IC_50 _> 45 nM [38].

The other ACT first-line treatment for uncomplicated *P*. *falciparum *malaria in Senegal is the combination of artemether-lumefantrine. Only 1% of the isolates presented reduced susceptibility to LMF. This prevalence did not rise in Senegal after the introduction of ACT. In 1996, 6% of isolates from Dielmo were resistant *in vitro *to LMF [31].

Six percent of isolates showed reduced susceptibility to QN, which is in accordance with previous studies in Dakar [24,36] and Dielmo [28,33,35]. Isolates with high IC_50 _to QN were already identified in 1984 [51]. However, a QN clinical failure was reported in a patient who spent two months in Dielmo in 2007 [52]. QN is often associated with DOX in the treatment of severe malaria in Dakar.

A prevalence of 12% of isolates with *in vitro *reduced susceptibility to DOX was observed in Dakar in 2009. The mean IC_50 _was similar to those estimated in Dielmo in 1998 [32,53]. Parasite populations in Senegal were distributed among three groups with low (4.9 μM), medium (7.7 μM) and high (17.7 μM) IC_50 _to DOX [53]. The slow activity of DOX *in vitro *has a delayed effect upon growth and requires prolonged incubation of parasites [32,54]. However, the standard 42-h test is still used to monitor DOX *in vitro *susceptibility.

The introduction of ACT in 2002 in Senegal did not induce a decrease in *P. falciparum *susceptibility to individual drug components, such as DHA, MDAQ and LMF. However, prevalence of *P. falciparum *isolates with reduced drug susceptibility increased for both MQ and DOX. Clinical failures with QN have been reported in Senegal. Additionally, isolates with high IC_50 _values for artemisinin derivatives have been described for Senegal and for clinical failures, or at least delayed parasite clearance times, have been described in Cambodia. For all of these reasons, an intensive surveillance of the *in vitro P. falciparum *susceptibility to anti-malarial drugs must be conducted in Senegal.

## Competing interests

The authors declare that they have no competing interests.

## Authors' contributions

BF, SD, KS, EB, YD, RB and BP carried out *in vitro *testing of drug susceptibility and drafted the manuscript. BD, KBF, PSM, FF and BW carried out diagnostic tests, monitored the patients, collected clinical and epidemiological data and drafted the manuscript. BF, CR, RB and BP analysed the data. All authors conceived and designed the study and read and approved the final manuscript.
